# The GRKs Reactome: Role in Cell Biology and Pathology

**DOI:** 10.3390/ijms22073375

**Published:** 2021-03-25

**Authors:** Preeti Kumari Chaudhary, Soochong Kim

**Affiliations:** Laboratory of Veterinary Pathology and Platelet Signaling, College of Veterinary Medicine, Chungbuk National University, Cheongju 28644, Korea; chaudharypreety11@gmail.com

**Keywords:** GRKs, GPCR, arrestins, G protein, cell biology, pathology

## Abstract

G protein-coupled receptor kinases (GRKs) are protein kinases that function in concert with arrestins in the regulation of a diverse class of G protein-coupled receptors (GPCRs) signaling. Although GRKs and arrestins are key participants in the regulation of GPCR cascades, the complex regulatory mechanisms of GRK expression, its alternation, and their function are not thoroughly understood. Several studies together with the work from our lab in recent years have revealed the critical role of these kinases in various physiological and pathophysiological processes, including cardiovascular biology, inflammation and immunity, neurodegeneration, thrombosis, and hemostasis. A comprehensive understanding of the mechanisms underlying functional interactions with multiple receptor proteins and how these interactions take part in the development of various pathobiological processes may give rise to novel diagnostic and therapeutic strategies. In this review, we summarize the current research linking the role of GRKs to various aspects of cell biology, pathology, and therapeutics, with a particular focus on thrombosis and hemostasis.

## 1. Introduction

G protein-coupled receptors (GPCRs) are the largest and most diverse class of signaling proteins that affect an incredible array of physiological processes throughout the human body. Therefore, one-third to half of all marketed drugs act by targeting GPCRs today. However, humans alone have nearly 1000 various GPCRs, and single ligand–receptor interaction leads to the divergent downstream signaling pathways in cells [1]. The continuous activation of these receptors may cause alteration in the physiological properties of the normal cell. Moreover, “druggable GPCRs” may develop drug resistance due to long-term exposure. These factors suggest that the long-held concept regarding GPCR signaling will no longer be adequate to design the next generation of therapeutic drugs. 

**Figure 1:**
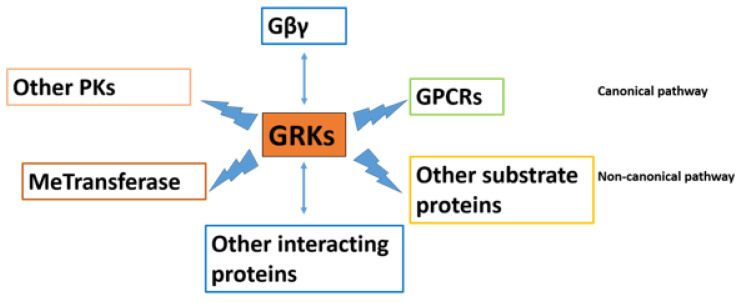
The complex G protein-coupled receptor kinases (GRKs) reactome. GRKs regulate diverse signaling pathways by interaction with proteins. GRKs interact with GPCRs in a canonical pathway. In addition, evidence has suggested that GRKs modulate signaling by interacting with other protein substrates in a non-canonical manner. They have a regulatory function with Gβγ subunits, other interacting proteins, MeTransferase, and other protein kinases (PKs).

Several mechanisms prevent the hyperactivation of GPCR signaling, among which GRKs and arrestins are the most important mechanism of terminating the GPCR activation, and these are therefore gaining popularity among researchers. GRKs initiate the kinase-dependent homologous desensitization of GPCRs by phosphorylating the activated GPCRs and allowing arrestin recruitment in a canonical pathway, thereby allowing cells to adapt to changing extracellular signals and prevent excessive signaling. There is a rapid loss of receptor responsiveness. GRKs also interact with other protein substrates and regulate various cellular interactions in a non-canonical manner (the interactions of GRKs with various proteins are depicted in Figure 1). Nevertheless, the profound data implicating GRKs in various cell biologies and pathologies suggest that GRK regulation can be considered as an important target of investigation in multiple aspects of diseases and their comorbidities, new diagnostic and novel therapeutics that would uncover improved treatments for diseases. Therefore, completely understanding the link between agonist-induced GPCR phosphorylation and the associated physiological effects is critical for the efficient targeting of cell signaling pathways in cell biology and diseases of high clinical importance.

Here, we will review the current research linking the role of GRKs to several aspects of cell biology and discuss the therapeutic strategies of targeting specific GRKs in different diseases, with a particular focus on thrombosis and hemostasis.

## 2. GRKs and Arrestins in GPCRs Family

### 2.1. GPCR Signaling Pathway; General Overview

GPCRs are seven-transmembrane (7-TM) receptors constituting a large protein family that connect intracellular and extracellular environments, and activate inside signal transduction pathways, and ultimately cellular response. There are five subfamilies of GPCRs: rhodopsin-like, secretin receptor, metabotropic glutamate, adhesion, and frizzled [2]. In terms of structure, each receptor is characterized by an extracellular region consisting of an N-terminus and three extracellular loops followed by a 7-TM α-helices region, and finally the intracellular region which includes three intracellular loops, an intracellular amphipathic helix, and the C-terminus [3]. Ligands such as light, proteins, ions, or small chemicals induce conformational changes in GPCRs and activate these receptors by coupling to heterotrimeric G protein complexes that consist of three subunits (Gα, β, and γ) [4,5]. Upon receptor binding of the ligand, the Gα subunit is activated and exchanges the active GTP-bound form in place of a GDP-bound form, which in turn triggers the dissociation of the Gα subunit from the Gβγ dimer. These dissociated subunits individually interact with the other specific intracellular effectors to continue the downstream GPCR signaling cascade. The βγ complex also interacts with downstream effectors including phosphoinositide 3-kinase γ (PI3Kγ) to activate GPCR-mediated signaling [6]. Further signaling depends on the type of G protein (G_q_/G11, G12/G13, Gi/Go/Gz, and Gs) each GPCRs encounters, followed by unique physiological responses [7].

### 2.2. The GRK and Arrestin Family

Although several proteins have been shown to interact directly with the cell surface 7-TM GPCRs [8], only two protein families besides heterotrimeric G proteins have the potential to interact with the activated conformation of 7-TM receptors: the GRKs and arrestins [9]. They are considered as the key modulators of important intracellular GPCR signaling cascades, as well as GPCR phosphorylation, desensitization, intracellular trafficking and resensitization [10,11,12,13]. GRKs belong to a subfamily of AGC-like kinases and thus share a conserved AGC sequence necessary for kinase activity [14]. There are three main types of GRKs based on the sequence homology: rhodopsin/visual kinases (GRK1 and GRK7), the β-adrenergic receptor kinases (GRK2 and GRK3), and the GRK4 subfamily (GRK4, GRK5, and GRK6). GRK2, 3, 5, and 6 are ubiquitously expressed in mammalian tissues, whereas GRK1 and 7 are exclusively expressed in the rod and cone cells, respectively, and GRK4 is expressed in testis, cerebellum, and kidney [15,16].

**Figure 2:**
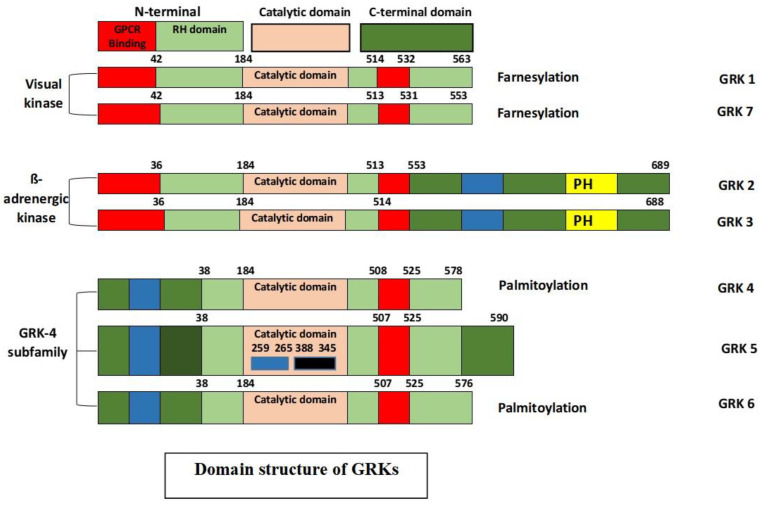
Structural domain distribution of the GRK isoforms. All GRKs (60–80 KDa) have a short N-terminal domain consisting of an α-N-terminal domain followed by an N-terminal regulator of G-protein signaling domain (RH: RGS homology) where the G protein-coupled receptor (GPCR) binds. A central catalytic domain for GRKs catalysis and a unique variable-length C-terminal domain (~105 to 230 amino acids) are important for receptor recognition. In contrast to other isoforms, the β-adrenergic kinase (GRK2 and GRK3) has a pleckstrin homology domain (PH) necessary for terminating Gβγ complex-related downstream signaling. The numbers above the domains represent amino acid residue as reported by Lodowski et al. [21].

These seven different genes share a similar basic protein structure, with an N-terminal, a central catalytic domain, and a unique variable-length C-terminal domain [17]. Each GRK has a highly conserved N-terminus and shares a considerable homology, which is believed to be critical for GPCR recognition, which renders it an important structure for the selective targeting of GPCRs. Apart from bearing homology, the N-terminal region displays several other motifs leading to an increase in its kinase catalytic activity. The C-terminal region contains phosphorylation sites, and key determinants of the cellular location and interaction with the lipids and/or membrane proteins, such as binding phosphatidylinositol 4,5-bisphosphate (PIP2) and Gβγ [18,19]. GRKs are also substrates for different kinases that take part in the modulation of kinase activity, localization, and stability [20]. The structure of GRKs is depicted in Figure 2. 

The arrestins are a small family of soluble proteins that specifically bind with the GRK-phosphorylated GPCRs and turn off intracellular signaling [22,23,24]. There are four members of the arrestin family. The expression of visual arrestins, arrestin 1 and arrestin 4, is restricted to retinal rod and cone cells, respectively. By contrast, non-visual arrestins, arrestin 2 and arrestin 3 (known as β-arrestin1 and β-arrestin 2), are expressed ubiquitously [25]. The amino acid sequences of the arrestin isoforms are 78% identical with most of the coding differences at the C terminus of the protein [26]. Mice lacking either arrestin 2 or arrestin 3 are shown to be viable [27,28], but arrestin 2/arrestin 3 double knockout mice die in utero [29]. Although it is not clear, arrestin isoforms might substitute each other functionally to some degree.

### 2.3. Cell Biology of GRKs and Arrestins in Signaling, Desensitization, Internalization, and Sorting of GPCRs

**Figure 3:**
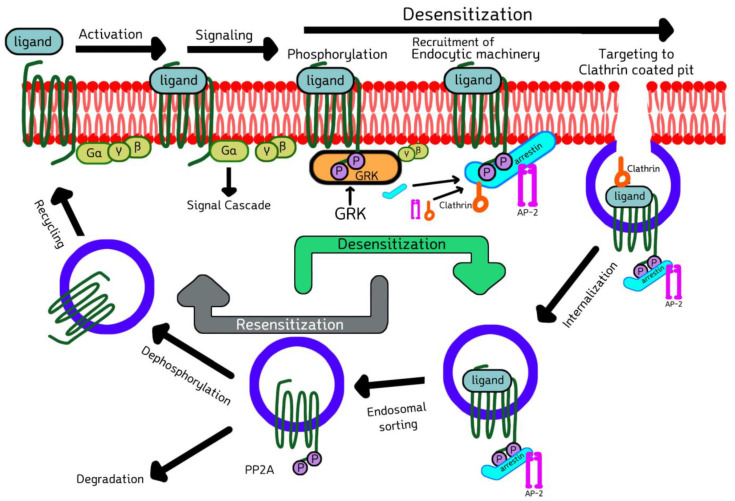
Model depicting GRKs and arrestins in the signaling, desensitization and internalization.The activated 7-TM receptor/GPCR is phosphorylated by GRK enabling arrestin recruitment and terminating G-protein coupling, finally desensitizing GPCR downstream signaling by mediating the arrestin–receptor complex to clathrin-coated pits. This promotes receptor internalization. The internalized receptor is finally sorted either by degradation or recycling. Refer to the text for a detailed mechanism. Modified from Mohan et al. [39].

Upon the ligand-binding activation of GPCR, G protein coupling, and signal transduction, GRK comes and phosphorylates the cytoplasmic serine and threonine residues of activated GPCR, enabling arrestin recruitment. The recruited arrestin then binds to the phosphorylated GPCR, inhibits further G-protein coupling and terminates the downstream signaling, a process termed desensitization [30]. These arrestin-bound receptor complexes are then targeted to clathrin-coated pits, where arrestin forms a multicomponent complex with the key elements of internalization machinery, such as clathrin [31] and adaptor protein-2 (AP-2) [32], and phosphoinositides, promoting endocytosis by budding inwardly from the membrane, thus resulting in receptor internalization. Finally, internalized GPCRs are sorted either into degradation or recycling compartments (Figure 3). This phenomenon was first demonstrated by the Kuhn group by showing that the phosphorylation of rhodopsin was necessary to stop its signaling [33]. Later, the Lefkowitz group reported that the β2-adrenergic receptor (β2-AR) is phosphorylated and desensitized by a cAMP-independent kinase [34]. It was verified that there is a general mode of two-step homologous GPCR desensitization where the active receptor is phosphorylated by one or more GRKs, and further arrestin binding stops the receptor signaling by direct competition with the G protein [35,36,37,38]. Various studies have been carried out showing that the GRKs’ kinase activity is regulated by interacting with multiple proteins, such as caveolin, RKIP, calmodulin, clathrin, actin, PI3K, Akt, etc.

There is a distribution of potential and established phosphorylation sites of GRKs in the selected GPCRs. These phosphorylation sites may be localized at the cytoplasmic region of the receptor. However, the potential GRK phosphorylation sites, their number, positions, and arrestin-binding capacity may vary among the same as well as various receptor subtypes. As the substitute of receptor desensitization, receptor internalization is often used as a readout for arrestin recruitment. However, these two processes are separate and have differential mechanistic requirements. In some cases, the phosphorylation of the receptor may not be necessary or have an insignificant role in binding arrestins to the receptor, as several arrestin-associated receptors do not require coated pits for internalization, while other arrestins might appear to only mediate receptor internalization, not desensitization. Usually, arrestin binding depends on the receptor’s C-cluster, which includes Thr307-Ser311 phosphorylation sites, and internalization is promoted by the phosphorylation of either the N-cluster that includes Ser286-Ser290 residues, or the C-cluster [40,41]. It has also been shown that some receptors do not recycle following internalization [42,43]. Therefore, there are multiple modes of not only the GRK-mediated phosphorylation of receptor subtypes, but also the succeeding arrestin recruitment, binding, and further downstream signaling, and thus the classical model does not necessarily apply to overall GPCR signaling.

Moreover, there are substantially more types of GPCRs compared to GRKs that might play a role in the interaction and phosphorylation of multiple GPCRs [44]. GRKs might exhibit substrate specificity with selective GPCRs, despite their domains’ structural similarity. Therefore, the biological function of each GRK isoform may differ remarkably [45]. Interestingly, GRKs can also substitute phosphorytable regions of their targeted GPCR when the interacting domain is mutated [46]. It has been shown that agonist-induced specific receptor conformation should be favorable for GRK binding and/or activation for a receptor to be phosphorylated by GRKs [47]. Among other things, GPCRs have plenty of promising serine and threonine phosphorylation sites. This suggests that these sites are not likely to be targeted all at once. It has been suggested that the order of phosphorylation may be either barcoded (where receptors responding to specific agonists will be phosphorylated by various GRKs at distinct sites, thus establishing a “barcode”), sequential (where a higher number of serine/threonine residues will be phosphorylated first) or hierarchical (where a particular sequence of serine and threonine will be preferentially targeted) [48,49,50,51]. However, the role of GRKs during GPCR activation and how GRKs recognize conformational changes in GPCR are still undetermined [52]. 

The cell type-dependent mechanisms of these GRKs on GPCRs, and how the specific phosphorylation of protein residues would control the consequent physiopathology of cell-biology and diseases, have just started to be untangled. The recent research in this area is discussed below.

## 3. The Role of GRKs and Arrestins in Regulation of GPCRs Family

### 3.1. The Role of GRKs in Immune Cells and Inflammation

Chemotaxis plays a significant role in inflammatory signaling by enabling the immune cells to appear at the site of inflammation. Chemokines-producing cells initiate the chemotaxis via chemokine receptor-mediated signaling (mainly GPCRs) through the integrated modulation of different steps, including receptor sensing, cell polarization, membrane protrusion, adhesion, or de-adhesion [53,54]. It has been demonstrated that chemokine GPCRs undergo desensitization upon constant stimulation of the receptors. Immune cells express high levels of GRK2, 3, 5, and 6, and are known to play a critical role in the modulation of immune cell response by involvement in the regulation of various cellular responses, such as scavenging, arrestins recruitment, signaling, and the desensitization of various chemokine receptors. The detailed function of GRKs in immune cells and inflammation is listed in Table 1.

Among various GRKs, GRK2 has been extensively studied in terms of chemotaxis and has been shown to negatively regulate chemotactic responses via canonical negative GPCR signaling [55,56,57]. It was shown to be involved in the regulation of cell type and stimulus-dependent immune cell migration [58,59]. Myeloid-specific GRK2 KO exhibited increased circulating neutrophils and macrophages mobilization to inflammatory sites, suggesting the desensitizing effect of GRK3 on chemokine receptors [60]. In malaria and sepsis, neutrophils showed an increased expression of GRK2 levels associated with decreased CXCR2 expression and reduced responses to IL-8 [61,62]. In contrast, it was shown that chemotactic responses in few other cell types are positively mediated by GRK2 [63]. GRK2 also plays a non-canonical role in cell motility, as it was shown to phosphorylate proteins such as ezrin, radixin and ERK1/2 in a kinase-independent manner [64,65]. Similarly, although in very few cell types, GRK5 has also been reported to regulate GPCRs such as CCR2 and CXCR4, which are critical in chemotaxis in both canonical and non-canonical manners [66,67,68,69]. Several chemokine receptors such as CXCR2, CXCR4, and LTB4 were found to be regulated by GRK6 in a canonical manner [70,71,72]. GRK6 was shown to modulate neutrophil and lymphocyte recruitment in vivo in various disease models [73,74,75]. The non-canonical role of GRK6 in immune cell chemotaxis is yet to be understood.

GPCRs have also been known to play a major role in the pathophysiological events in sepsis, a severe inflammatory response, via cardiovascular, immune, and coagulatory responses. It was shown that GRK2 and GRK5 play a notable role in the pathogenesis of human sepsis by regulating neutrophil chemotaxis, and modulate the outcomes of septic shock via the NF-κB1p105-TPL2-MEK-ERK pathway [61,76,77,78,79]. However, these GRKs have no role in immune cell infiltration, the presence of bacteria, or patient survival. It was shown that GRK5 deficiency leads to decreased cytokine levels, decreased thymocyte apoptosis and immune suppression, and reduced plasma corticosterone levels, leading to sepsis-induced mortality even in the presence of antibiotics in both endotoxemia and polymicrobial sepsis models [77,80].

All these results indicate the important roles of GRKs in immune cell chemotaxis and inflammatory signaling, and indicate that the identification of the small-molecule compounds that regulate GRK isoforms could be a plausible therapeutic approach in inflammatory disease management.

### 3.2. The Role of GRKs in Cardiovascular Diseases

Although significant improvements have been made with the drugs targeting GPCRs to treat cardiovascular patients, the treatments remain insufficient. The disease-causing effect of GPCRs can initially be mitigated by negative feedback via GRKs, as GRKs have widely established themselves as having a primary role in modulating GPCR signaling. GRKs have been thoroughly studied as possible diagnostic or therapeutic targets in the cardiovascular system. GRK activity and expression were shown to be altered in various cardiovascular system diseases, including congestive heart failure, hypertension, myocardial infarction, and cardiac hypertrophy [95]. GRK2, GRK3, and GRK5 play a well-established role in the progress of diseases of the cardiovascular system [96,97]. GRK2 and GRK5 have been associated with heart failure in humans and their increased expression/activity was shown to induce β-adrenergic receptor desensitization [98,99]. GRK2 expression was shown to be enhanced in hypertension, cardiac hypertrophy, and myocardial infarction, and the inhibition of GRK2 was observed to have beneficial effects on these diseases [100,101,102,103]. GRK5 has been found to regulate the vascular endothelial growth factor (VEGF) receptor in the endothelial cells of the coronary artery [104]. The overexpression of GRK2 and GRK5 in vivo has been shown to decrease adrenergic receptor-induced myocardial contractility and cardiac output, which were counteracted by GRK2, GRK3, and GRK5 inhibition [96,98,103,105]. It was shown that hypertensive patients display enhanced GRK2 activity and protein expression with no any changes in GRK5, GRK6, PKA or arrestins, suggesting disease-dependent variation in GRK2 [101]. GRK2 and GRK5 have also been associated with the development of atherosclerosis [60,66]. 

Overall, studies indicate that GRK inhibition has direct advantageous effects in cardiovascular disease conditions by promoting survival signals, but the pathophysiological role of GRKs in cardiovascular diseases is still incompletely understood. Therefore, increasing the understanding of GRKs’ molecular and cellular processes is of critical importance in order to improve therapeutic strategies.

### 3.3. The Role of GRKs in Neurodegeneration and Autoimmune Diseases

GRKs have been shown to play a role in the pathogenesis of neurodegenerative diseases, including Alzheimer’s disease (AD), multiple sclerosis (MS), and Parkinson’s disease [106,107,108]. GRK2 levels were increased in the human brain and this was reported to serve as a marker for early hypoperfusion-induced brain damage in AD patients [109]. During hypoxic-ischemic injury, GRK2 was shown to exacerbate brain damage via p38-dependent TNFα production [83], regulate the metabotropic glutamate receptor function and expression implicated in the pathogenesis of AD and MS, and cause neurodegeneration via the over-activation of group I mGLuTs [110,111]. GRK2 was also shown to reduce inflammatory hyperalgesia by inhibiting microglial activation via the inhibiting of p38-dependent TNFα production and PGE2-mediated pathways [112]. Nociceptor function leading to chronic pain was shown to ause a long-lasting neuroplastic change with the inhibition of GRK2 levels [113]. Similarly, GRK5 was shown to be involved in the regulation of the desensitization of muscarinic receptors, specifically M2 and M4 [114]. GRK5-deficient mice have been shown to increase the incidence of AD-like pathology. Mice-deficient in GRK6 were shown to develop an autoimmune disease, such as arthritis and colitis, by modulating the infiltration of immune cells [73,75,115].

Together, these studies reveal that GRKs do play a critical role in the pathogenesis of neurodegenerative as well as autoimmune disease, and thus can be targeted for therapeutic development.

### 3.4. The Role of GRKs in Cancer

The evolving evidence shows that GPCRs are being used as an early diagnosis biomarker, as GPCR signaling plays integral roles in regulating various aspects of cancer biology, including vascular remodeling, invasion, cell proliferation, apoptosis and migration, by regulating cancer-associated signaling pathways [116,117]. Developing drugs targeting GPCR and its signaling pathway has potential as a new therapeutic strategy for the treatment of cancers. As GRKs have established themselves as a negative regulator of GPCR activity, several studies have displayed the function of GRKs in cancer progression in a cell type-dependent manner [118,119]. An overview of the established roles of GRKs in different types of cancer is summarized in Table 2.

Briefly discussing the role of GRKs in cancer, GRK1 and GRK7 were shown to play a role in embryogenesis, while interacting indirectly with Rho GDP-dissociation inhibitor (RhoGDI) and phosphodiesterase γ (PDEγ), both of which are shown to be abnormally regulated in cancer [153,154]. GRK1/7 might play a role in the development of cancer-related retinopathy in lung cancer patients by interacting with a calcium-binding protein called recoverin [152]. GRK2, but not GRK5, is more highly expressed in differentiated thyroid carcinoma than normal thyroid tissue, and is involved in the rapid desensitization of thyroid-stimulating hormone receptor (TSHR), which desensitization can be further inhibited by GRK5. A study found out that cancer cell proliferation was increased due to the activation of TSHR in thyroid carcinoma [120]. GRK2 has been shown to negatively regulate the insulin-like growth factor-1 receptor (IGF1-R) signaling involved in proliferation and migration by decreasing cyclin in human hepatocellular carcinoma HepG2 cells [121]. GRK2 also plays an important role in suppressing cell cycle progression, regulating early growth response 1 (EGR1) expression, and the progression of breast, gastric, and skin cancer by modulating GPCR signaling [122]. Furthermore, clinical studies have revealed a correlation between high GRK2 expression and a high tumor (T) stage, as well as the poor survival rates of patients suffering from pancreatic cancer. Interestingly, nerve growth factor (NGF) in combination with GRK2 promotes opioid receptors phosphorylation whilst amplifying the pain, and when treated with anti-NGF therapies, there was significant relief in cancer bone pain by mediating the outcome of GRK2 and arrestins. GRK2 acted as a negative regulator of the chemokine receptor CXCR4 (C-X-C chemokine receptor type 4), which is responsible for mediating metastasis and is generally applied as a patient prognosis indicator [155]. Several studies showed that GRK3 inhibited breast cancer metastasis by regulating CXCR4 signaling [44,139]. GRK3 was abnormally expressed in oral squamous carcinoma cells probably via the activation of the β2-adrenergic receptor [128]. Arterial angiotensin type 1 (AT1) and dopamine D receptor signaling have been shown to be modulated by GRK4 [156,157]. Several studies found that GRK4α/β desensitizes the follicle-stimulating hormone receptor (FSHR) and its expression was significantly lower in malignant ovarian granulosa cells compared to benign granulosa cells, suggesting the crucial function of GRK4α/β in the course of ovarian granulosa cell transformation [135]. GRK5 expression is associated with a worse prognosis in patients that are suffering from stage II-IV glioblastoma. GRK2 and GRK5 are known to work oppositely in thyroid cancer [120]. GRK5 could be responsible for the direct phosphorylation of P53 tumor suppression in U2OS and Saos-2 osteosarcoma cells degradation promotion, consequently inhibiting tumor cell apoptosis [145]. A positive association was found between GRK6 and Ki-67 expression, pathological disease stage, metastasis, and survival rate in the patients with hepatocellular carcinoma, and GRK6 was hypothesized as a biomarker for the early diagnosis of hepatocellular carcinoma [148].

### 3.5. The Role of GRKs in Thrombosis and Hemostasis

Platelets develop from bone marrow, and are anucleated discoid cells of approximately 2 to 4 µm in diameter [158]. The resting platelet consists of an organelle zone formed by alpha granules, dense granules, lysosomal granules, and it glycogen granules, and contains more than 800 different proteins involved in major platelet functions [159,160]. Platelets are primarily responsible for the aggregation process and contribute to coagulation. During a vascular lesion, platelets are activated, and their granules release factors that are involved in the coagulation. 

The activation of platelets is important in hemostasis and thrombosis [161]. Initial platelet activation signals are greatly amplified by a series of rapid positive feedback loops, enabling robust platelet recruitment and thrombus stabilization. The detailed mechanism of platelet activation has been reviewed elsewhere [162]. In brief, upon the vascular injury or high shear stress of the blood flow, circulating platelets come into contact with the exposed sub-endothelium, causing the release, generation, or exposure of agonists, which in turn can activate platelets, resulting in the onset of the thrombus formation process. Platelets are activated by adhesion to adhesive proteins (von Willebrand factor (vWF), collagen) or soluble agonists (ADP, thrombin, thromboxane A_2_ (TxA_2_), serotonin, epinephrine) via their respective adhesion receptors or GPCRs, respectively [163,164,165].

The key initiators of the platelet activation at the sites of endothelial damage are the adhesion receptors, while GPCRs play a critical and central role in platelet activation and thrombus formation [165]. Interestingly, despite significant variations in functions and downstream signaling pathways, important platelet receptors share a lot of similarities in their signal transduction mechanisms. For example, the complex of adhesion receptors GP Ib–IX–V, glycoprotein VI (GPVI), and integrins all involve Src family kinases (SFKs), PI3Ks, and the immunoreceptor tyrosine-based activation motif (ITAM) signaling pathway, while GPCRs involve phospholipase C (PLC), Ca^2+^, diacyl gylcerol (DAG), protein kinase C (PKC), and the PI3Ks signaling pathway. These receptor-specific platelet activations signal pathway cross-talk and merge into common signaling events that trigger platelet shape change, granule secretion, and TxA_2_ generation, activating integrin α_IIb_β_3._ Ligand binding to integrin α_Iib_β_3_ causes platelet adhesion and aggregation, leading to the activation of “inside-out” signaling. Inside-out signaling triggers the “outside-in” signaling, leading to the spreading of platelets, additional granule content secretion and clot retraction, finally stabilizing the platelet adhesion and aggregation. 

It has been reported that human platelets can become refractory to activation after major surgery, possibly leading to an increased risk of post-surgical bleeding [166]. This clearly demonstrates that, like platelet activation and aggregation, the desensitization mechanism plays an equally critical role in regulating platelet responsiveness. There are several mechanisms by which GPCR- and G-protein-dependent signaling is turned off in various cells, including GRKs- and arrestin-induced desensitization, RGS protein-regulated turning off, and the internalization of receptors by forming endosomes. Platelets express GRKs and arrestins, and it is well known that the signaling of platelets largely occurs through GPCRs. Platelet GPCRs regulate platelet function by coupling to their respective G-protein, such as G_q_-coupled PARs, thromboxane A_2_ receptor (TP), P2Y_1_, 5HT; G_i_-coupled P2Y_12_; G_z_-coupled adrenergic receptors; and G_s_-coupled IP, upon the stimulation of platelets with various agonists, including ADP, TxA_2_, thrombin, serotonin, epinephrine, and prostacyclin. 

Platelet GPCRs are clinically relevant GPCRs, which are targeted by multitudinous drugs of therapeutical importance for bleeding disorders or anticoagulation. Considering the critical roles of GRKs in GPCR functions in other cells, very little is known about the regulation and mechanisms of GPCR signaling and GPCR desensitization by GRKs in platelets. Very recently, we and others have unveiled the interesting features of the GRK system in platelet GPCR signaling. Using GRK6 KO mouse platelets, we showed that the platelet aggregation and dense granule secretion induced by GPCR agonists, including 2-MeSADP, U46619 (TxA_2_ analog), AYPGKF, and thrombin, is significantly potentiated compared to WT platelets [167]. However, GPVI agonist collagen-related peptide (CRP)-induced platelet aggregation and dense granule secretion are not affected in the GRK6-deficient platelets, indicating that GRK6 does play a role in the regulation of P2Y_1_, P2Y_12_, TPα, and PARs-mediated signaling in platelets, and does not regulate non-GPCR-mediated platelet activation. We found that the U46619-induced aggregation response curve shifts left, but the extent of potentiation was not as significant as it was for the other GPCR agonists. This may be due to the shorter C-terminus of the platelet TxA2 receptor TPα that has lesser phosphorylatable serine residues, leading to the decreased affinity of GRK6 towards it compared to other GPCRs in platelets. Similar to our study, it was demonstrated that the GRK phosphorylation sites for β1AR and β2AR are in their C-termini, whereas β3AR lacks GRK targets and it has a very short C-terminus [168]. GRK6 also affects GPCR-mediated integrin αIIbβ3 activation and P-selectin expression in platelets [167]. Most recently, Chen et al. showed that GRK6 has a role in ADP-induced P2Y_12_ receptor desensitization, but not P2Y_1_ receptor desensitization in platelets [169]. Hardy et al. also reported that GRK2 and GRK6 mediate P2Y_12_, but not P2Y_1_, receptor desensitization in astrocytoma cells [170]. In contrast to their study, we observed that GRK6 is critical in regulating both ADP-induced P2Y_1_ and P2Y_12_ receptor desensitization. GRK’s kinase activity has been shown to vary according to cell type, and the same GRK isoform may have a role in desensitization and activation, or show no response at all to a specific receptor. 

Interestingly, in contrast to ADP-induced aggregation, GRK6 is not involved in serotonin-induced G_q_-coupled 5HT_2A_ receptor and epinephrine-induced G_z_-coupled α_2A_ adrenergic receptor-mediated platelet activation, although the co-stimulation of serotonin and epinephrine mimics the ADP-induced P2Y_1_ and P2Y_12_ receptor-mediated platelet activation pathway, further suggesting that the kinase activity of GRKs varies with varying ligands, receptors and proteins. Since platelet GPCRs have been the topmost target for anti-thrombotic drug development, research should be done as such, instead of following the “model” concept and predicting the functional mechanism. We also found that the re-stimulation of platelets with ADP and AYPGKF could restore platelet aggregation in GRK6-deficient platelets, suggesting the role of GRK6 in the desensitization of ADP and PAR4 receptors [167]. Moreover, G_q_- and G_i_-mediated signaling events were regulated by GRK6 in platelets. GRK6 -/- mice are more susceptible to thrombosis, and thus, GRK6 plays a critical role in platelet function in vivo [167].

It remains unknown whether other GRK isoforms except GRK6 phosphorylate the platelet GPCRs when stimulated with various agonists, and whether or how this phosphorylation will change the behavior of the receptor. It is important to know the potential phosphorylation sites, and evidently the number of absolutely phosphorylated sites, in order to establish the receptor’s behavior in platelets. Neither the putative phosphorylation sites nor their specific function is determined in platelet GPCRs. Which GRK isoform is involved in each phosphorylation event during platelet signaling remains unknown. It would be fascinating to see whether or not the platelet agonists promote the receptor conformation in the same way for GRK binding and activation, subsequent receptor phosphorylation, and arrestin recruitment/binding, or whether there is the involvement of some other mechanisms. 

Arrestins have been shown to play crucial roles in terminating GPCR-mediated signaling in various cells. Platelets also express arrestin 2 and arrestin 3. Despite the importance of arrestins in GPCR-mediated signaling, the mechanism of GPCR desensitization by arrestins in platelets has not been clearly elucidated yet. Li et al. first showed that the PAR4 and ADP receptor signaling in platelets is differentially regulated by arrestin 2, as only thrombin-, but not ADP-, induced PI3K-Akt phosphorylation and fibrinogen binding was arrestin 2-dependent [171]. Later, Schaff et al. reported that neither arrestin 2 nor arrestin 3 deficiency altered platelet activation, suggesting that arrestins are not involved in platelet GPCR desensitization [172]. They further reported that the deletion of arrestin 2 in mice, but not arrestin 3, is critical for thrombus formation. In contrast, very recently, a study demonstrated the negative regulatory role of arrestin 3 downstream of PAR4- and P2Y_12_-mediated signaling pathways in mouse platelets [173]. It is not clear which arrestin plays what functional role in platelets, and further study is required to determine the functional differences of arrestin 3 versus arrestin 2 in the regulation of GPCR signaling and the molecular basis of GPCR desensitization in platelets. 

Evaluating the functional significance of the individual GRK and β-arrestin isoforms in platelets, and characterizing the novel mechanisms involved in GPCR desensitization and trafficking in platelets using knockout mice for each isoform, will help us to seek a novel approach to developing drugs in the area of vascular pathobiology. 

#### Contribution of Platelet Beyond Thrombosis and Hemostasis

Platelets were believed to have only hemostatic activity. In recent years, scientific research and technology have come up with a new perspective on platelets and their functions due to their abundant granular content of growth factors (GFs), cytokines, and other biological modulators that, upon release, can affect wound healing, inflammation, angiogenesis, stem cell migration, and cell proliferation. These factors also have a paracrine effect in different cell populations, including mesenchymal cells, osteoblasts, fibroblasts, and endothelial cells, which are all involved in various pathophysiological processes [174,175]. 

Platelets have been shown to play a significant role in inflammation and immunity. Activated, adherent platelets at the vascular injury site can recruit leukocytes to the injury or inflammatory sites, and largely mediate the signaling involved in initial and sustained platelet–leukocyte interactions by regulating receptors including the epithelial neutrophil-activating peptide, glycoproteins, growth-related oncogenes, interleukins, intercellular adhesion molecules, junctional adhesion molecules, platelet-activating factor, and P-selectin glycoprotein ligands. Platelets are capable of changing their surface expression and release their granule content through their TLRs, P-selectin, RANTES, TGF-β, etc., thereby engaging different endothelial and immune cells, including neutrophils, monocytes, eosinophils, B cells and T cells, dendritic cells, natural killer cells, etc., and potentially leading to the activation of the innate and adaptive immune responses. Similarly, platelets can increase the endothelial permeability and mediate leukocyte trafficking to the inflamed endothelium by activating endothelial cells [176]. Besides this, platelet-originating TxA_2_ form a positive feedback loop upon platelet activation that facilitates the further release of their substantial repertoire of stored cytokines, including IL-1α, IL-1β, Macrophage Inflammatory Protein (MIP)-1α, CD40 ligand (CD40L), and polyphosphate (polyp), which have been shown to contribute to multiple inflammation-related diseases. Importantly, it was shown that platelets play a major role in netosis.

Platelets express diverse mediators, such as PDGF, TGF, and VEGF, that integrate various cascades governed by multiple cytokines, and are released after activated platelets become entrapped within the fibrin matrix. These mediators have been shown to play crucial roles in remodeling and wound healing by stimulating the mitogenic responses required for tissue repair [177]. 

In the early phase of infection, platelets can limit parasite growth by killing plasmodium falciparum through the release of platelet factors [178,179]. Platelets can interact with various strains of bacteria including the staphylococci family, *Neisseria gonorrhoeae*, *Porphyromonas gingivalis*, and *Helicobacter pylori* [180,181,182]. During bacterial infections, platelets actively mediate the host response through interactions with circulating leukocytes. Platelet α-granules contain various antimicrobial compounds such as platelet connective tissue-activating peptide 3 (CTAP-3), platelet basic protein, thymosin β-4 (Tβ-4), and fibrinopeptide (A and B), which are known to target various bacterial organisms [183,184]. Platelets are also known to interact with various types of viruses through TLR2, IL-1β, and VEGF. Recently, platelets were shown to play a major role in the pathological consequences of COVID-19 as well [185]. Therefore, platelets are beneficial in infections, but consistent viral or bacterial infections can cause arterial thrombosis or venous thromboembolism, a process termed immune thrombosis, leading to cardiovascular disease. Recently, it has been suggested that antiplatelet medications may lower mortality rates related to infections and sepsis.

Platelets also participate in neural diseases associated with pathogen-induced and sterile inflammation, including AD, MS, and migraine, suggesting its role in CNS inflammatory and immune response [186,187]. It has been shown that IgE stored in platelet α-granules upon release has the potential to amplify allergic responses [188]. It was shown that there is a significant interconnection between the activation of platelets and eosinophils, platelets and leukocytes in asthma, and lung allergic inflammation, respectively [189,190,191]. Overall, platelets play important roles in diverse inflammatory diseases, and targeting platelet signaling could be a promising approach to modify platelet responses to inflammation.

It is now generally accepted that platelets play a major role in the early stage of endothelial disturbance in the atherosclerotic process and subsequent CVD [192]. As the markers for predicting the clinical fate of CVD, the molecules activating platelets have been suggested, and may be targeted to minimize thrombosis and atherosclerosis. The variability in platelet activation would also affect the formation of atherosclerosis, thereby affecting CVD. Conversely, limiting platelet activation and aggregation, and inhibiting the molecules involved, can regulate platelet interactions, thrombosis, and CVD. 

Much of the recent data has indicated that platelets play a serious role in the pathogenesis of malignant cancers. Using animal models, it has been demonstrated that platelets make a major contribution to tumor cell proliferation, metastasis, and tumor angiogenesis in various types of cancers, such as carcinomas of the breast, colon, lung, and ovary, as well as in melanoma [193,194]. It has been shown that circulating platelet properties contribute to some hallmarks of cancer, including resisting cell death, inducing angiogenesis, metastasis, and evading immune detection, assisting cancer stem cells, and sustaining proliferative signals. Besides this, patients with metastatic cancer have higher chances of thrombosis. Platelets interact with tumor cells, resulting in platelet activation, P-selectin expression, and the development of platelet–tumor microthrombi, which may protect tumor cells from the innate immune system [195]. Therefore, some have developed anti-platelet antibodies to recognize the activated platelet and then further fragment the platelet, potentially reducing metastatic potential [196,197]. Aspirin was also shown to be associated with a reduced risk of distant metastasis in patients with cancer, particularly of colorectal origin [198].

In conclusion, since platelets are involved in other disease conditions beyond thrombosis and hemostasis, the identification of GRKs and their functional receptors in platelets will serve to design novel drug targets and receptor blockers, contributing to the improved treatment of patients with platelet-associated diseases. Therefore, current research is focused on possible therapeutic interventions targeting platelet activation, desensitization, or its surface receptors.

## 4. The Implications of GRKs in Pharmacology

The chronic or acute use of drugs that target GPCR is associated with an increasing level of GRK expression. A study was carried out that investigated GPCR-targeted drug tolerance in the brain, which demonstrated that an increase in GRK expression could be responsible for drug resistance [199,200]. Likewise, there is a correlation between several pathological conditions such as heart failure, depression, Alzheimer’s disease and Parkinson’s disease, and modulated endogenous GRK expression [108,201,202,203]. Due to scientists’ continuous efforts to gain more knowledge about GPCR/GRK signaling biology, new treatment strategies have been developed for diseases by targeting GRKs. Presumably, the development of highly selective GRK inhibitors will either target their specific kinase domains or reduce the expression of GRK by using selective RNA aptamers [204]. So far, none of the effective GRK inhibitors have been permitted for use in clinical practice [205]. GRKs belong to the AGC kinases subfamily whose kinase domains are nearly identical in structure; there is a possibility of cross-reactivity in the nonselective GRK inhibitors with other AGC kinases [206]. Nevertheless, Takeda Compound 103A, which is a highly selective GRK2 inhibitor that has been shown to inhibit GRK2 activity 50-fold compared to other AGC kinases, has been developed by Takeda Pharmaceuticals [207]. Meanwhile, other highly selective GRK-targeted drugs, for instance paroxetine, GSK180736A, balanol, Takeda Compound 101, and sanigivamycin, which target GRK2, GRK3, GRK5, GRK2, and GRK6, respectively, are also being extensively investigated [208]. Even though these drugs are rather selective for GRKs, all of them demonstrate cross-reactivity with other kinases that limit the drug’s therapeutic potential. Each GRK isoform plays a variety of roles in disease progression due to their structural heterogeneity. Investigating the divergent roles of specific isoforms may allow us to develop potential drugs that would guarantee a selective inhibitory effect on specific isoforms for better disease control. The selective knockdown of specific GRKs and understanding the pharmaceutical properties using various cellular ligands would allow us to design and develop drugs more practically and efficiently, and unveil novel concepts in therapy.

## 5. Conclusions

In conclusion, GRKs along with arrestins play a central role, as well as controlling hundreds of unique GPCRs and their signaling in almost every facet of cell biology and diseases, making it difficult to fully perceive their mechanism of cellular signaling. GRKs act as important regulators to prevent cells from hyper-stimulation, and could directly modulate the physio-pathological functions of cell biology. As such, GRKs help in maintaining homeostasis by mediating inter- and intra-cellular communication in response to the surrounding environment. As several GPCR-targeted disease treatments are becoming more attractive topics, GRKs could be propitious molecular targets for controlling GPCR responsiveness. It is important to identify the full mechanism involved in GPCR–GRK interactions. Which GRK isoform and how it is involved in each phosphorylation event, the specific effects of these phosphorylation events on subsequent arrestin binding, and cell type-dependent GPCR desensitization/internalization, are still aspects that are yet to be understood. It would be interesting to investigate if GPCR agonists fail to uphold the receptor conformation that is suitable for GRK binding or activation, and consequent receptor phosphorylation as well as arrestin recruitment, or whether they function via some other mechanism.

## Figures and Tables

**Table 1:** GRK isoforms and their established functions in immune cells and inflammation.

GRK Isoform	Interacting Partner(s)	Associated Signalling Pathway/Cellular Response	References
GRK2	NF-kB p105 subunit and inhibitor (IkB-α) phosphorylation	TLR4-induced and Tumor Necrosis Factor-α (TNF-α) pathways	[79,81,82]
	p38 phosphorylation Raf1, MEK1, ERK2, RhoA, RKIP, GIT	P38 mitogen-activated protein kinases (MAPK) pathways Extracellular signal-regulated kinase (ERK) pathways	[83,84,85]
	Serine-threonine kinase Akt phosphorylation	Akt-nitric oxide (NO) pathways	[86,87]
	Ezrin/radixin/moesin phosphorylation	Actin cytoskeleton	[64,65]
	ADP ribosylation factor (ARF)-specific GTPase-activating proteins (GIT)	Focal adhesion dynamic	[63,88]
	Histone deacetylase 6 (HDAC6) phosphorylation	Microtubules network	[89]
	Heat shock protein 90 (Hsp90)	Regulation of GRK expression	[90]
	Receptor-regulated Smads (R-Smads) phosphorylation	Transforming growth factor β (TGF-β) pathways	[91,92]
GRK3	HSP90	Regulation of GRK expression	[90]
GRK5	ERM (moesin phosphorylation)	Actin cytoskeleton	[69]
	GIT1	Regulation of receptor endocytosis	[88]
	HSP90, HSP70	Regulation of GRK expression and CXCR4 endocytosis	
	NF-kB p105 subunit and IkB-α phosphorylation	TLR4-induced and TNF-α pathways	[90,91]
	Src Tyrosine kinase	GRK phosphorylation and neutrophils exocytosis	[93]
GRK6	HSP90	Regulation of GRK expression	[90,94]

**Table 2:** An overview of established roles of GRK isoforms in various types of cancer.

GRK Subtype	Type of Cancer	Interacting Partner (s)	Molecular Mechanism	Function	Biological Model	References
GRK2	Thyroid carcinoma	TSHR	ND	Decrease proliferation through rapid desensitization	Differentiated thyroid carcinoma patients and cell lines	[120]
	Hepatocellular carcinoma cell	IGFI-R		Decrease proliferation and migration		[121]
	Human hepatocellular carcinoma (HepG2)	IGFI-R		Decrease cell cycle progression		[122]
	Pancreatic cancer	N/A	ND	--T-stage and poor survival rate, increased proliferation	Pancreatic carcinoma patients, ductal adenocarcinoma patients and cell lines	[123]
	Breast carcinosarcoma	NGFR		Decrease bone cancer pain		[124]
	Kaposi’s sarcoma-associated herpesvirus infected tumor cell	CXCR2	Desensitization and AKT signaling	Decrease migration and invasion	Patients and cell lines	[125]
	Basal breast cancer with Her-2 amplification/infiltrating ductal carcinoma	Her-2/ER-α		Increase the promoting of mitogenic, anti-apoptic activities- survival and progression		[126]
	Luminal and basal breast cancer	HDAC6/Pin1	AKT/ERK cascades	Increase sensitivity of breast cancer cells to traditional chemotherapeutic treatment	Invasive ductal carcinoma patients, cell lines orthotopic and xenograftmouse models	[127]
	Breast cancer	CXCR4	Desensitization and signaling	Decrease metastasis	Breast cancer patients, cell lines orthotopic mouse models	[128]
	Human gastric carcinoma cell line (MKN-45)	H2 receptor		--poor differentiation		[129]
	Human breast cancer	N/A		Increase tumor growth and decrease angiogenesis		[130,131]
	Prostrate	ND		Differentiation	Adenocarcinoma patients	[132]
	Prostrate	ND	ND	ND	Neuroendocrine prostrate and metastatic castrastion-resistant prostate cancer patients	[133]
	Glioblastoma	ND	ND		Mesenchymal glioblastoma patients.	[134]
GRK2/4	Ovary	ND	ND		Granulosa cell cancer patients	[135]
GRK2/5/6	Gastric cancer (SSTW-2)	recoverin		--tumor progression, metastasis		[136]
GRK2/6	melanoma	Melanocortin 1 receptor		--determinant for skin cancer		[137]
GRK3	Breast cancers (MDA-MB-231, MDA-MB-468	CXCR4		Decrease metastasis increase migration	Breast cancer patients, cell lines orthotopic mouse models	[138]
	Prostate cancer (PC3)	N/A	Downmodulation of angiogenesis inhibitors	Increase metastasis, tumor progression, angiogenesis	Metastatic castration-resistant prostate cancer patients, cell lines and orthotopic mouse models	[139]
	Retinoblastoma (Y-79)	CRFI receptor		Increase stress adaptation		[140]
	Oral squamous carcinoma	β2-adrenergic receptor		--tumor malignancy and invasion		[128]
	Glioblastoma	CXCR4 desensitization and signaling	desensitization and signaling	Increased proliferation	Classical Glioblastoma patients	[134]
GRK4	Ovarian malignant granulosa cell tumor	FSHR		--benign and malignant transformation in tumor development		[135]
	Breast cancer	Arrestin2 receptor	Mediated ERK & JNK signaling	Increase proliferation	Ductal carcinoma patients and cell lines	[141]
GRK5	glioblastoma	N/A		Proliferation rate and WHO grade	Glioblastoma multiform patients and cell lines	[142]
	Thyroid carcinoma	TSHR	TSHR desensitization and signaling	Decrease proliferation through slow desensitization, increase proliferation	Differentiated thyroid carcinoma patients	[120]
	Prostate cancer (PC3)	Cyclin D1	G2/M progression	Decrease proliferation, cell cycle	Cell lines and xenograft mouse tumors	[143]
	Prostate cancer (PC3, DU145, LNCaP)	Moesin	Moesin phosphorylation	Decrease migration, invasion Increase cell adhesion	Cell lines and xenograft mouse tumors	[69]
	Prostate cancer	N/A		Increase tumor growth, invasion, and metastasis		[144]
	Osteosarcoma (U2OS, Saos-2)	P53	Phosphorylation and degradation	Decrease cell apoptosis and radiosensitivity	Cell lines	[145]
	Colon	PGE2	Desensitization and signaling	Increased proliferation	Cell lines	[146]
	Kaposi’s sarcoma	KSHV-GPCR	Desensitization and signaling	Increased proliferation	Cell lines	[147]
GRK6	Heptocellular carcinoma	N/A		--proliferation maker in early diagnosis		[148]
	Hypopharyngeal squamous cell carcinoma (FaDu)	Methyl transferase	Methylation of GRK6	--cancer progression Decrease invasion		[149]
	Medulloblastoma	CXCR4/ EGFR/ PDGFR-Src		Increase migration		[143]
	Lung cancer	CXCR2		Decrease cancer development		[150]
	Lung	ND	ND	Decreased survival	Adenocarcinoma patients	[151]
	Medullo-Blastoma	CXCR4	Desensitization and signaling	Increased migration	Medulloblastoma patients and cell lines	[143]
	Myeloma	STAT3	phosphorylation	Increased survival	Primary multiple myeloma patients and cell lines	[94]
GRK1/7		recoverin		--cancer-associated retinopathy		[152]

N/A: not provided by the research, --: has association with.

## Abbreviations

GRK	G protein-coupled receptor kinase
GPCR	G protein-coupled receptor
7-TM	Seven-transmembrane
PI3K	Phosphoiositide 3-kinase
PIP2	Phosphatidylinositol 4:5-bisphosphate
RGS	Regulator of G protein signaling domain
PH	Pleckstrin homology
AP-2	Adaptor protein-2
β2-AR	β2-adrenergic receptor
VEGF	Vascular endothelial growth factor
AD	Alzheimer’s disease
MS	Multiple sclerosis
RhoGDI	Rho GDP-dissociation inhibitor
PDE	Phosphodiesterase
TSHR	Thryoid-stimulating hormone receptor
IGF1-R	Insulin-like growth factor-1 receptor
EGR1	Early growth factor 1
NGF	Nerve growth factor
CXCR	Chemokine receptor
AT1	Angiotensin type 1
FSHR	Follicle-stimulating hormone receptor
vWF	Von Willebrand factor
TxA_2_	Thromboxane A_2_
PLC	Phospholipase C
DAG	Diacyl glycerol
PKC	Protein kinase C
TP	Thromboxane A_2_ receptor
GPVI	Glycoprotein VI
SFKs	Src family kinases
ITAM	Immunoreceptor tyrosine-based activation motif
CRP	Collagen-related peptide
GF	Growth factor
MIP	Macrophage inflammatory protein
